# Liver Injury in Critically Ill and Non-critically Ill COVID-19 Patients: A Multicenter, Retrospective, Observational Study

**DOI:** 10.3389/fmed.2020.00347

**Published:** 2020-06-23

**Authors:** Saiping Jiang, Rongrong Wang, Lu Li, Dongsheng Hong, Renping Ru, Yuefeng Rao, Jing Miao, Na Chen, Xiuhua Wu, Ziqi Ye, Yunzhen Hu, Minghua Xie, Minjuan Zuo, Xiaoyang Lu, Yunqing Qiu, Tingbo Liang

**Affiliations:** ^1^Department of Pharmacy, The First Affiliated Hospital, College of Medicine, Zhejiang University, Hangzhou, China; ^2^Department of Pharmacy, Xixi Hospital of Hangzhou, Hangzhou, China; ^3^Department of Pharmacy, First People's Hospital of Yuhang District, Hangzhou, China; ^4^Public Service Platform for the Evaluation of Innovative Drug Property, Hangzhou, China; ^5^State Key Laboratory for Diagnosis and Treatment of Infectious Disease, Collaborative Innovation Center for Diagnosis and Treatment of Infectious Diseases, Zhejiang Provincial Key Laboratory for Drug Clinical Research and Evaluation, The First Affiliated Hospital, College of Medicine, Zhejiang University, Hangzhou, China; ^6^Department of Hepatobiliary and Pancreatic Surgery, The First Affiliated Hospital, College of Medicine, Zhejiang University, Hangzhou, China

**Keywords:** Incidence, risk factors, liver injury, COVID-19, disease severity

## Abstract

**Background:** Liver injury commonly occurs in patients with COVID-19. There is limited data describing the course of liver injury occurrence in patients with different disease severity, and the causes and risk factors are unknown. We aim to investigate the incidence, characteristics, risk factors, and clinical outcomes of liver injury in patients with COVID-19.

**Methods:** This retrospective observational study was conducted in three hospitals (Zhejiang, China). From January 19, 2020 to February 20, 2020, patients confirmed with COVID-19 (≥18 years) and without liver injury were enrolled and divided into non-critically ill and critically ill groups. The incidence and characteristics of liver injury were compared between the two groups. Demographics, clinical characteristics, treatments, and treatment outcomes between patients with or without liver injury were compared within each group. The multivariable logistic regression model was used to explore the risk factors for liver injury.

**Results:** The mean age of 131 enrolled patients was 51.2 years (standard deviation *[SD]*: 16.1 years), and 70 (53.4%) patients were male. A total of 76 patients developed liver injury (mild, 40.5%; moderate, 15.3%; severe, 2.3%) with a median occurrence time of 10.0 days. Critically ill patients had higher and earlier occurrence (81.5 vs. 51.9%, 12.0 vs. 5.0 days; *p* < 0.001), greater injury severity (*p* < 0.001), and slower recovery (50.0 vs. 61.1%) of liver function than non-critically ill patients. Multivariable regression showed that the number of concomitant medications (odds ratio [OR]: 1.12, 95% confidence interval [CI]: 1.05–1.21) and the combination treatment of lopinavir/ritonavir and arbidol (OR: 3.58, 95% CI: 1.44–9.52) were risk factors for liver injury in non-critically ill patients. The metabolism of arbidol can be significantly inhibited by lopinavir/ritonavir *in vitro* (*p* < 0.005), which may be the underlying cause of drug-related liver injury. Liver injury was related to increased length of hospital stay (mean difference [MD]: 3.2, 95% CI: 1.3–5.2) and viral shedding duration (MD: 3.0, 95% CI: 1.0–4.9).

**Conclusions:** Critically ill patients with COVID-19 suffered earlier occurrence, greater injury severity, and slower recovery from liver injury than non-critically ill patients. Drug factors were related to liver injury in non-critically ill patients. Liver injury was related to prolonged hospital stay and viral shedding duration in patients with COVID-19.

**Clinical Trial Registration:** World Health Organization International Clinical Trials Registry Platform, ChiCTR2000030593. Registered March 8, 2020.

## Introduction

Since December 2019, a newly recognized acute respiratory illness, now officially named coronavirus disease-19 (COVID-19), has become widespread globally and accounts for considerable human morbidity and mortality over 200 countries, areas, and territories worldwide (1–4). The novel coronavirus is identified and designated as severe acute respiratory syndrome coronavirus 2 (SARS-CoV-2) (5) by the International Committee on Taxonomy of Viruses. As of May 30, 2020, more than five million COVID-19 cases had been diagnosed worldwide, and almost 360,000 deaths had been reported (2). COVID-19 has been officially declared a pandemic by the World Health Organization (6) due to the ongoing outbreak globally.

The common clinical manifestations of COVID-19 include fever, cough, and shortness of breath (4, 7, 8). According to the latest epidemiological studies, ~16–53% of patients with COVID-19 experienced different degrees of liver injury (4, 7–13), and some patients have developed severe liver injury. The coagulant function abnormality induced by liver injury may cause serious bleeding, especially in critically ill patients who are receiving continuous renal replacement or extracorporeal membrane oxygenation. Liver function deterioration can lead to liver failure and even death. Therefore, liver injury in patients with COVID-19 needs close attention. Although some studies have reported the incidence of liver injury in patients with COVID-19 (8, 14, 15), there are limited data describing the course of liver injury occurrence, such as liver injury onset, progression, and recovery, during an entire hospitalization period, particularly in patients with different disease severity.

Studies on the causes and risk factors of liver injury during SARS-CoV-2 infection are still limited and controversial. According to current research, liver injury in COVID-19 is associated with several main factors, such as SARS-CoV-2 infection, treatment with potentially hepatotoxic drugs, virally induced cytotoxic T cells, and dysregulated innate immune response (16). Moderate microvesicular steatosis and mild lobular activity are observed in the liver tissue of patients with COVID-19 (17). A preliminary study indicates that SARS-CoV-2 may directly bind to ACE2-positive cholangiocytes to dysregulate liver function. However, systematic study of the causes and the risk factors of liver injury in specific populations, such as critically ill and non-critically ill patients with COVID-19, is still lacking. Additionally, little is known about the correlations between liver injury and some important clinical outcomes, such as length of hospital stay and duration of SARS-CoV-2 shedding. Therefore, a further in-depth study is needed.

Here, we conducted a multicenter, retrospective, observational study to explore liver injury in critically ill and non-critically ill patients with COVID-19 in Zhejiang Province, China. This study aims to reveal the course of occurrence, risk factors, and correlations with clinical outcome of liver injury in specific COVID-19 populations. Our study may be helpful in understanding the pathogenesis of liver injury in patients with COVID-19, preventing liver injury, and optimizing individual therapeutic treatment.

## Materials and Methods

### Study Design and Participants

This retrospective observational study was conducted in three tertiary hospitals designated to treat patients with COVID-19 in Zhejiang Province, China. The study was launch by the First Affiliated Hospital of Zhejiang University, which is a university-affiliated tertiary hospital with 2m500 beds and over 100,000 discharged patients per year. SARS-CoV-2 infection was confirmed using real-time polymerase chain reaction (4, 7) by the local designated hospitals. From January 19, 2020, to February 20, 2020, patients diagnosed with COVID-19 were enrolled in this study. Patients were excluded on the basis of the following criteria: (1) pregnancy in women, (2) age under 18 years, and (3) liver injury on admission.

This study followed the statement of Strengthening the Reporting of Observational Studies in Epidemiology and was approved by the Ethics Committee of the First Affiliated Hospital, College of Medicine, Zhejiang University (Reference Number: 2020IIT[71]). The data were anonymous, and the requirement for informed consent was waived. The study was registered at the World Health Organization International Clinical Trials Registry Platform (ChiCTR2000030593) on March 8, 2020.

### Data Collection

The clinical electronic medical records were reviewed, and epidemiological, clinical, demographic, laboratory, and outcome data were collected for all included patients. A standard case report form was used to record data, including sex, age, chronic medical illness, laboratory data, systemic antiviral agents (i.e., lopinavir/ritonavir, arbidol, fapilavir, and darunavir/cobicistat), potentially hepatotoxic concomitant drugs (18–21) (i.e., corticosteroids, quinolones, statins, immunosuppressive drugs, and non-steroidal anti-inflammatory drugs [NSAIDs]), number of concomitant drugs, length of hospital stay, and duration of viral shedding. Clinical data were followed up until March 10, 2020. Topical drugs were not included in concomitant medications. Missing data were obtained by direct communication with doctors responsible for the treatment of the patient and their families. All data were verified by three researchers.

### Definitions

The severity of COVID-19 was defined as non-severe (mild or moderate pneumonia), severe (severe pneumonia), and critically ill during admission in accordance with the diagnostic and treatment guidelines for SARS-CoV-2 pneumonia of the Chinese National Health Committee (version 6) (22). In accordance with the severity of COVID-19, the patients were divided into non-critically ill (non-severe and severe disease severity) and critically ill groups. Non-severe cases included patients with mild and moderate COVID-19. The clinical symptoms of mild cases were mild, and there was no sign of pneumonia on imaging. Moderate COVID-19 refers to fever and respiratory symptoms with radiological findings of pneumonia. Severe COVID-19 refers to cases meeting any of the following criteria: (1) respiratory distress, (2) oxygen saturation, and (3) arterial partial pressure of oxygen (PaO2)/fraction of inspired oxygen (FiO2) ≦300 mmHg or cases with chest imaging that showed obvious lesion progression within 24–48 h >50%. Critically ill refers to cases meeting any of the following criteria: (1) respiratory failure necessitating mechanical ventilation, (2) shock, and (3) combination with organ failure and admission to an intensive care unit.

Liver injury was defined as any increase above the normal range for alanine aminotransferase (ALT), aspartate aminotransferase (AST), alkaline phosphatase (ALP), or total bilirubin (TBL). The degree of liver injury was classified as mild, moderate, or severe (Table 1) in accordance with the Common Terminology Criteria for Adverse Events (version 5) (23). Mild, moderate, and severe liver injuries were defined as the occurrence of adverse event grades 1, 2, and 3 (i.e., the increase in ALT, AST, ALP, or TBL, Table 1), respectively. The recovery rate of liver function was defined as the decrease in the number of patients with liver injury at discharge divided by the number of patients with liver injury during treatment.

**Table 1:** Definition of liver injury.

**Indicators**	**Liver injury^**a**^**	**Mild liver injury^**b**^**	**Moderate liver injury^**c**^**	**Severe liver injury^**d**^**
ALT	>1 ULN	>1–3 ULN	>3–5 ULN	>5 ULN
AST	>1 ULN	>1–3 ULN	>3–5 ULN	>5 ULN
ALP	>1 ULN	>1–2.5 ULN	>2.5–5 ULN	>5 ULN
TBIL	>1 ULN	>1–1.5 ULN	>1.5–3 ULN	>3 ULN

a, b, c, d *Liver injury, mild, moderate, and severe liver injury were defined as the occurrence of any of the listed abnormal liver function indicators in the corresponding columns, respectively*.

Viral clearance was defined as the presence of two consecutive negative results with qPCR detection over an interval of 24 h.

### Metabolic Interactions Between Lopinavir/Ritonavir and Arbidol *in vitro*

The metabolic interactions between lopinavir/ritonavir and arbidol was tested in human hepatic microsomes. The metabolic reaction was performed in 0.1 ml incubation mixture containing 0.5 mg microsome protein. The reaction was started by adding 1 mM nicotinamide adenine dinucleotide phosphate and terminated by adding 0.3 ml acetonitrile after metabolism for 60 min. The sample was mixed and centrifuged at 15,000 rpm for 15 min. An aliquot of 5 μL supernatant was injected into the LC-MS/MS system. The final concentrations of arbidol were 5, 20, and 50 mM, and those of lopinavir/ritonavir were 5/1.25, 20/5, and 50/12.5 mM.

### Statistical Analysis

Statistical tests were performed using SPSS19.0 (www.spss.com) and R 3.5.1 (R Core team, www.r-project.org). Continuous variables were presented as mean (standard deviation [*SD*]) or median (interquartile range [IQR]) and compared between and within non-critically ill and critically ill groups by using the Student's *t*-tests or the Mann–Whitney *U*-test, as appropriate. Categorical variables were presented as frequency (percentage) and assessed using the Pearson χ^2^ or Fisher's exact test (cell size <5).

The occurrence time of liver injury was defined from the time a patient was admitted to hospital until liver injury occurred. The occurrence time of liver injury was portrayed by the Kaplan–Meier plot and compared between patients in critically ill and non-critically ill groups with a log-rank test. Liver injury after admission and at discharge, abnormal liver function indicators, and recovery rate of liver function were compared between the critically ill and non-critically ill groups. Univariable and multivariable logistic regression models were used to explore the risk factors for liver injury. Demographic data, laboratory test indicators, disease severity, antiviral agents, and potentially hepatotoxic concomitant drugs were investigated. The factors that showed a significant association (95% confidence interval [CI]: does not include one) after univariate logistic regression analysis were entered into the multivariable logistic regression analysis. Clinical outcomes (i.e., length of hospital stay and duration of viral clearance) were compared between patients with or without liver injury within each group. *p* < 0.05 was considered statistically significant.

## Results

### Demographic and Clinical Characteristics

**Figure 1:**
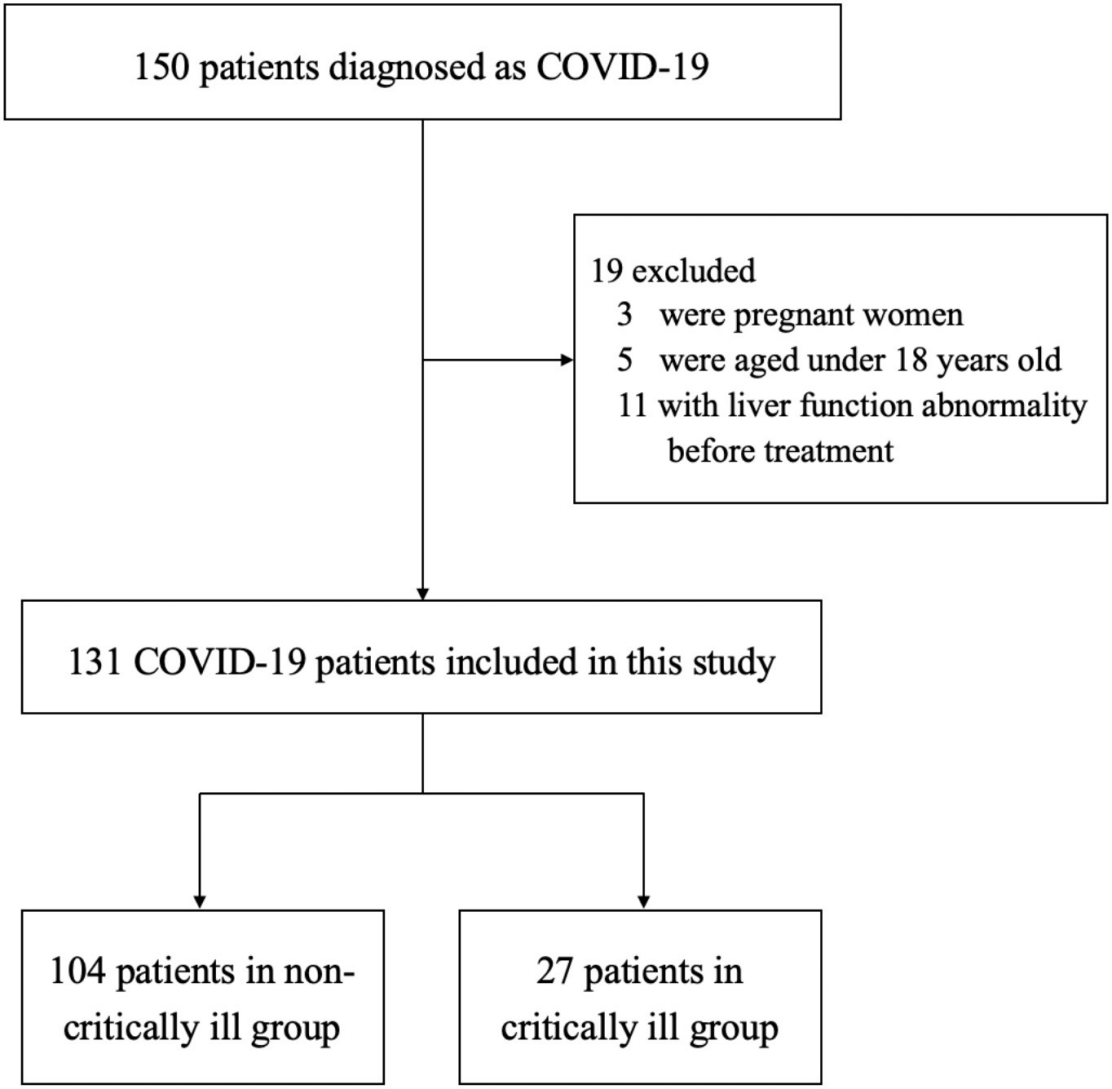
Study flowchart.

The demographic and clinical characteristics of the patients are shown in Table 2. Nineteen ineligible patients were excluded, and the clinical data of 131 patients with confirmed COVID-19 were collected (Figure 1). The sample sizes of included patients were 62, 47, and 21 patients from the three hospitals, respectively. Out of 131 patients, 70 (53.4%) were male. The mean age was 51.2 years (range: 19–96 years). Of these patients, cardiovascular and cerebrovascular diseases (*n* = 37, 28.2%) and endocrine system disease (*n* = 22, 16.8%) were the most common coexisting conditions. The severity of COVID-19 was categorized as non-severe in 75 (57.3%) patients, severe in 29 (22.1%) patients, and critically ill in 27 (20.6%) patients. During the treatment, each patient received an average of nine (IQR: 6–12)) concomitant medications. A total of 66 (50.4%) patients received glucocorticoids, 34 (26.0%) patients received quinolones, and nine (6.9%) patients received NSAIDs. As for antiviral agents, 77.9% of patients received the combination treatment of lopinavir/ritonavir and arbidol, and 15.3% of patients received darunavir/cobicistat-based therapy.

**Table 2:** Demographics and clinical characteristics of 131 enrolled COVID-19 patients.

**Characteristics**	**All patients** **(*n* = 131)**	**Non-critically ill** **(*n* = 104)**	**Critically ill** **(*n* = 27)**	***p***
**Age, Years**				
Mean (*SD*)	51.2 (16.1)	47.2 (13.3)	67.0 (16.2)	<0.001
Range	19–96	19–88	37–96	
**Sex**				0.135
Male	70 (53.4%)	52 (50.0%)	18 (66.7%)	
Female	61 (46.6%)	52 (50.0%)	9 (33.3%)	
BMI	23.2 (3.2)	23.0 (3.2)	23.9 (3.3)	0.198
**Chronic medical illness**				
Cardiovascular and cerebrovascular diseases	37 (28.2%)	17 (16.3%)	20 (74.1%)	<0.001
Endocrine system disease	22 (16.8%)	14 (13.5%)	8 (29.6%)	0.078
Digestive system disease	5 (3.8%)	3 (2.9%)	2 (7.4%)	0.274
Neurological disorders	4 (3.1%)	4 (3.8%)	0 (0%)	
Immune system	2 (1.5%)	2 (1.9%)	0 (0%)	
**Laboratory results**				
Leucocytes (× 10^9^/L)	6.9 (4.2)	6.0 (3.0)	8.1 (5.5)	0.009
Neutrophils (%)	71.4 (17.2)	68.0 (15.3)	83.5 (9.5)	<0.001
Lymphocytes (× 10^9^/L)	2.0 (4.8)	2.3 (5.2)	1.1 (1.8)	0.241
Haemoglobin (g/L)	135.8 (17.9)	136.9 (18.1)	127.5 (17.2)	0.017
Platelets (× 10^9^/L)	205.3 (76.7)	208.8 (77.3)	190.9 (84.5)	0.295
C-reactive protein (mg/L)	26.4 (35.9)	20.2 (32.7)	45.8 (38.5)	0.001
Total bilirubin (μmol/L)	11.9 (7.7)	12.0 (8.1)	11.3 (6.3)	0.677
Direct bilirubin (μmol/L)	5.5 (3.4)	5.3 (3.4)	5.6 (3.0)	0.678
Alanine aminotransferase (ALT, μmol/L)	22.5 (12.6)	24.1 (16.6)	18.4 (6.7)	0.084
Aspartate aminotransferase (AST, μmol/L)	24.5 (8.9)	24.8 (10.7)	26.7 (6.8)	0.382
Alkaline phosphatase (ALP, U/L)	67.1 (17.3)	68.2 (16.2)	62.6 (20.8)	0.460
Albumin (g/L)	39.4 (5.4)	40.5 (5.0)	36.1(5.1)	<0.001
Serum creatinine (μmol/L)	73.6 (30.9)	69.6 (19.7)	91.2 (53.4)	0.001
Number of concomitant medications	9 (IQR, 6–12)	8 (IQR, 6–12)	15 (IQR, 11–19)	<0.001
**Concomitant medications**				
Glucocorticoids	66 (50.4%)	40 (38.5%)	26 (96.3%)	<0.001
Quinolones	34 (26.0%)	23 (22.1%)	11(40.7%)	0.082
NSAIDs	9 (6.9%)	7 (6.7%)	2 (7.4%)	>0.999
Statins	4 (3.1%)	4 (3.8%)	0 (0%)	
Immunosuppressive agents	3 (2.3%)	3 (2.9%)	0 (0%)	
**Antiviral agents**				
Lopinavir/ritonavir + arbidol^a^	102 (77.9%)	90 (86.5%)	13 (48.1%)	<0.001
Darunavir/cobicistat-based therapy^b^	20 (15.3%)	8 (7.7%)	12 (44.4%)	<0.001
Others	9 (6.9%)	6 (5.8%)	2 (7.4%)	0.668

Data are n (%) and mean (SD) unless specified otherwise.

a Dose of lopinavir/ritonavir: 400 mg/100 mg twice daily; Dose of arbidol: 200 mg three times daily.

b *Dose of darunavir/cobicistat: 800 mg/150 mg once daily*.

The baseline characteristics of patients in critically ill and non-critically ill groups were significantly different (*p* < 0.05) for the parameters age, prevalence of cardiovascular and cerebrovascular diseases, laboratory data (i.e., leucocytes, neutrophils, albumin, serum creatinine, and C-reactive protein), and treatments (i.e., number of concomitant medications and use of glucocorticoids and antiviral agents).

### Incidence and Characteristics of Liver Injury

**Figure 2:**
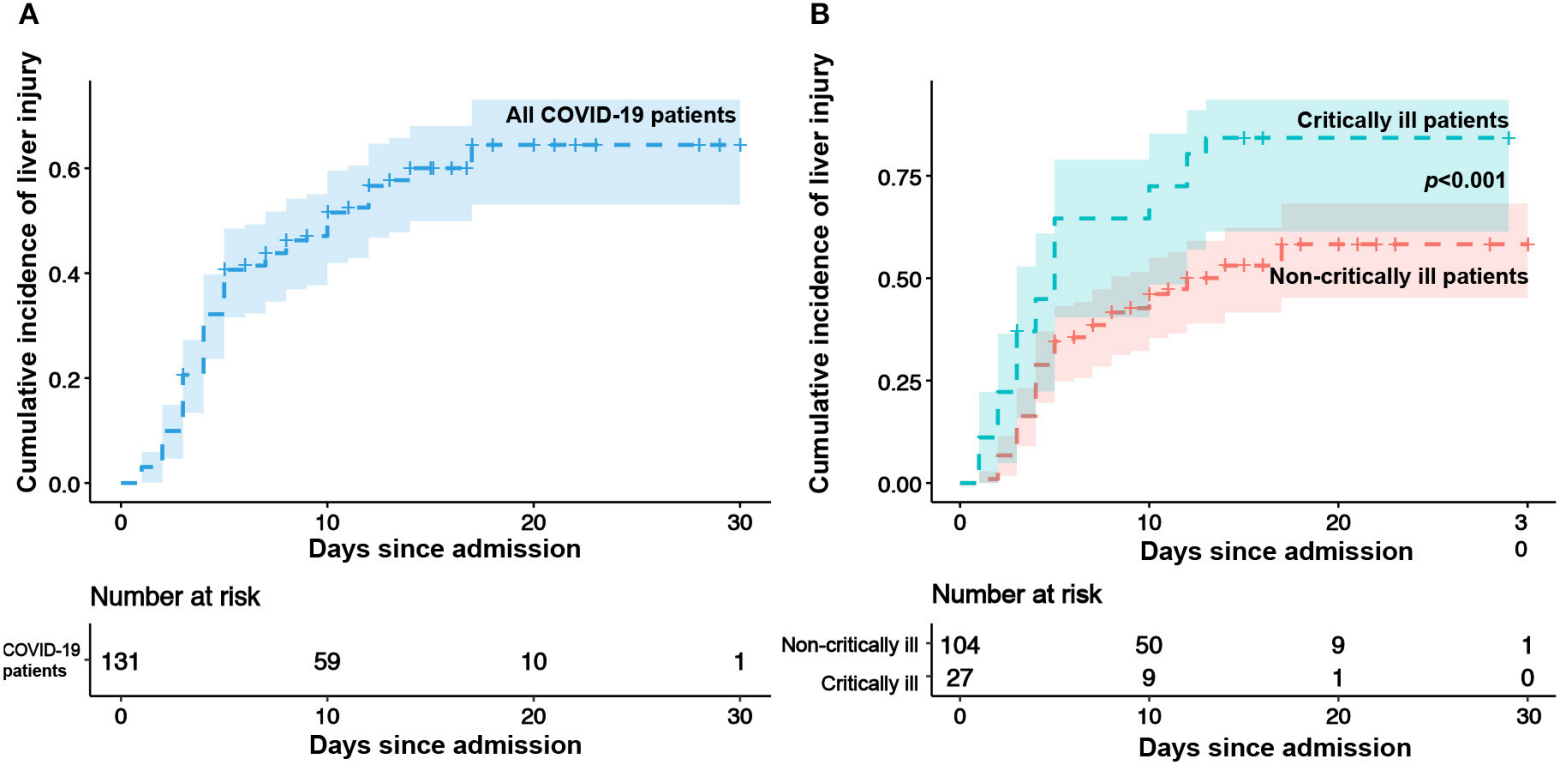
Kaplan–Meier survival curve for liver injury. **(A)** Kaplan–Meier curve for liver injury in all enrolled patients. Shaded area shows point-wise *SD*. **(B)** Kaplan–Meier survival curves for liver injury in non-critically ill and critically ill patients.

The incidence of liver injury over the study period is shown in Figure 2. During the treatment, 76 patients (58.0%) had liver injury (mild, 40.5%; moderate, 15.3%; severe, 2.3%, Table 3). The median occurrence time of liver injury was 10.0 days. The percentage of liver injury was reduced to 24.4% at discharge, and liver function returned to normal levels in 57.9% of patients with liver injury. However, none patients with severe liver injury returned to the normal liver function at discharge (Table 3).

**Table 3:** Characteristics of liver injury in enrolled patients during hospitalization.

**Liver function**	**All patient** **(*n* = 131)**	**Non-critically ill** **(*n* = 104)**	**Critically ill** **(*n* =2 7)**	***p***
Liver injury	76 (58.0%)	54 (51.9%)	22 (81.5%)	0.006
Degree of liver injury				<0.001
Mild	53 (40.5)	42 (40.4%)	11 (40.7%)	
Moderate	20 (15.3%)	10 (9.6%)	10 (37.0%)	
Severe	3 (2.3%)	2 (1.9%)	1 (3.7%)	
Abnormal ALT	45 (34.4%)	31 (29.8%)	14 (51.9%)	0.032
Abnormal AST	41 (31.3%)	28 (26.9%)	13 (48.1%)	0.034
Abnormal ALP	7 (5.3%)	5 (4.8%)	2 (7.4%)	0.633
Abnormal TBL	43 (32.8%)	36 (34.6%)	7 (25.9%)	0.392
Liver injury at discharge	32 (24.4%)	21 (20.2%)	11 (40.7%)	0.027
Recovery after liver injury	44/76 (57.9%)	33/54 (61.1%)	11/22 (50.0%)	0.374
Recovery after different degrees of liver injury				
Mild	35/54 (66.0%)	26/42 (61.9%)	9/11(81.8%)	0.296
Moderate	9/19 (45.0%)	7/10 (70.0%)	2/10 (20.0%)	0.060
Severe	0/3 (0.0%)	0/2 (0.0%)	0/1 (0.0%)	

*Data are n (%) unless specified otherwise. P-values were calculated by comparing the distribution of patients within the non-critically ill and critically ill groups by Pearson's χ^2^ or Fisher's exact test (cell size <5)*.

The Kaplan–Meier survival curves for liver injury in different groups are presented in Figure 2. The incidence of liver injury was significantly different between patients in critically ill and non-critically ill groups (Figure 2, median: 12.0 day vs. 5.0 days, *p* < 0.001) over the study period. As shown in Table 3, 81.5% of the patients in the critically ill group developed liver injury, compared with 51.9% in the non-critically ill group. The severity of liver injury in the critically ill group was greater (*p* < 0.001) than that in the non-critically ill group. ALT and AST levels were more commonly elevated in critically ill patients (*p* < 0.05) than in the non-critically ill group, whereas no statistical difference was observed in the abnormal ALP and TBL levels (Table 3). The recovery rate of liver function in the non-critically ill group was higher than that in the critically ill group (61.1 vs. 50.0%).

### Risk Factors for Liver Injury

Univariable and multivariable logistic regression models were used to explore the risk factors for liver injury in the critically ill and non-critically ill groups. However, given the limited sample size in the critically ill group, the statistical power was insufficient, and the multivariable logistic regression model was not conducted in this group. The comparison between patients with or without liver injury in the critically ill group showed that the patients with liver injury had lower lymphocyte numbers, received more concomitant medications, and had higher serum creatinine levels on admission, but the differences were not statistically different (Supplementary Table S1).

In the non-critically ill group, the univariate logistic analyses showed that the combination treatment of lopinavir/ritonavir and arbidol and the number of concomitant medications were significantly associated with liver injury (Table 4). Patients developing liver injury received more concomitant medications (mean difference [MD]: 2.05, 95% CI: 0.36–3.74, *p* = 0.018) compared with patients with normal liver function (Supplementary Table S2). The percentage of patients receiving lopinavir/ritonavir combined with arbidol was higher than that of patients without liver injury (92.6 vs. 80.0%, Supplementary Table S2). Correlations among age, sex ratio, BMI, disease severity, concomitant medicines (i.e., glucocorticoids, quinolones, NSAIDs, statins, and immunosuppressive agents), and risk for liver injury were not observed in this group (Table 4).

**Table 4:** Univariate and multivariable logistic analysis for risks factors for liver injury in non-critically patients.

**Covariate**	**Univariate analysis**		**Multivariable analysis**	
	**OR**	**95%CI**	**OR**	**95%CI**
**Demographics characteristics**				
Age (year)	0.982	0.953–1.012		
Female (*n*, %)	0.629	0.287–1.359		
BMI (kg/m^2^)	0.977	0.864–1.104		
Chronic medical illness (*n*, %)	0.555	0.229–1.312		
Cardiovascular and cerebrovascular diseases (*n*, %)	0.443	0.142–1.273		
Endocrine system disease (*n*, %)	0.656	0.201–2.038		
Severe disease severity (*n*, %)	1.198	0.507–2.868		
**Treatment-related factors**				
Treatment course of antivirus (day)	1.043	0.956–1.142		
Lopinavir/ritonavir + arbidol (*n*, %)	3.929	1.652–9.966	3.584	1.442–9.523
Darunavir/cobicistat-based therapy (*n*, %)	0.282	0.040–1.294		
Number of concomitant medications (number)	1.118	1.020–1.236	1.121	1.049–1.212
Glucocorticoids (*n*, %)	1.222	0.554–2.719		
Quinolones (*n*, %)	1.268	0.501–3.288		
NSAIDs (*n*, %)	1.253	0.263–6.642		
Statins (*n*, %)	0.296	0.014–2.396		
Immunosuppressive agents (*n*, %)	1.885	0.175–41.310		
**Laboratory results**				
Normal Leucocytes (*n*,%)	1.710	0.771–3.851		
Normal Neutrophils (*n*, %)	1.046	0.470–2.337		
Normal lymphocytes (*n*, %)	0.778	0.333–1.792		
Normal haemoglobin (*n*, %)	0.916	0.275–2.963		
Normal platelets (*n*, %)	2.035	0.575–8.208		
Normal serum creatinine (*n*, %)	0.998	0.979–1.018		

*NSAIDs, Nonsteroidal anti-inflammatory drugs*.

After the multivariable regression analysis, the combination treatment of lopinavir/ritonavir and arbidol and the number of concomitant medications were determined to be independent risk factors for liver injury. The patients who received the combination treatment of lopinavir/ritonavir and arbidol had 3.58 times the odds (95% CI: 1.44–9.52) of liver injury than patients who did not receive the aforementioned treatment. For every increase in concomitant medication, the odds of liver injury increased by 12.1% (95% CI: 4.9%−21.2%, Table 3).

### Metabolic Interactions Between Lopinavir/Ritonavir and Arbidol *in vitro*

**Figure 3:**
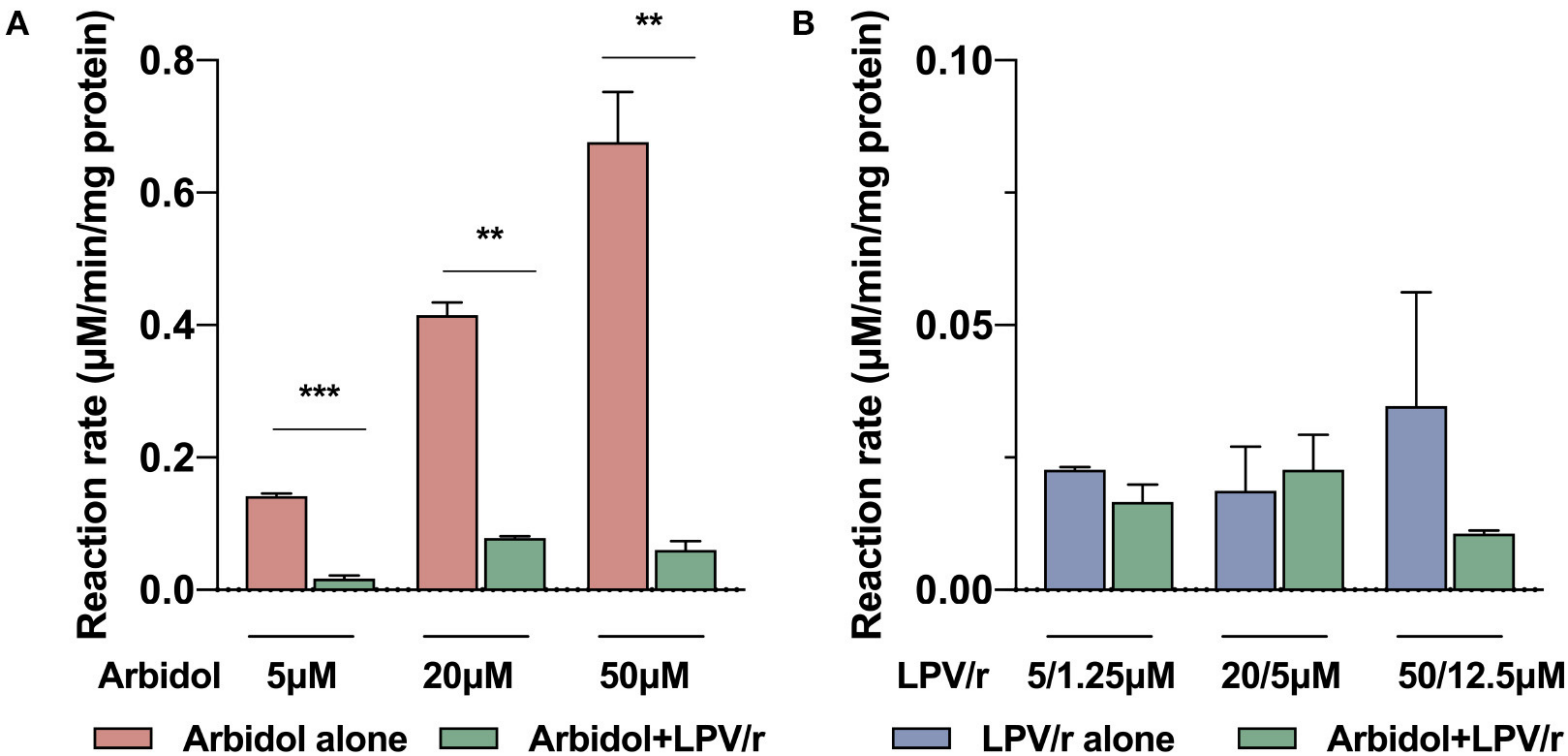
The metabolic interaction between lopinavir/ritonavir and arbidol *in vitro*. **(A)** The metabolic inhibition of lopinavir/ritonavir on arbidol *in vitro*. **(B)** The metabolic inhibition of arbidol on lopinavir/ritonavir *in vitro*. LPV/r: lopinavir/ritonavir. **p < 0.01, ***p < 0.001.

Lopinavir/ritonavir combined with arbidol was shown to be a risk factor of liver injury. We inferred that the metabolic interaction between arbidol and lopinavir/ritonavir may increase drug concentrations and may thus lead to a higher risk of liver injury. The metabolic interactions were tested in human hepatic microsomes *in vitro*, and the results showed that the metabolism of arbidol can be significantly inhibited after exposure to different concentrations of lopinavir/ritonavir (*p* < 0.005, Figure 3), whereas arbidol had no effect on the metabolism of lopinavir/ritonavir (*p* > 0.05, Figure 3).

### Correlations Between Liver Injury and Clinical Outcomes

The average length of hospital stay was 16.6 (*SD*: 5.7) days and was statistically longer in patients with liver injury than in patients without liver injury (MD: 3.2, 95% CI: 1.3–5.2). The mean duration of viral clearance in patients with liver injury was 13.6 days, which was 3 days longer than that in patients with normal liver function (95% CI:1.0–4.9). Within the non-critically ill and critically ill groups, the length of hospital stay and duration of viral clearance tended to increase in patients developing liver injury (Table 5).

**Table 5:** Treatment outcomes of enrolled patients.

	**All patients** **(*n* = 131)**	**Non-critically ill** **(*n* =1 04)**	**Critically ill** **(*n* = 27)**
Length of hospital stay (days)	16.6 (5.7)	15.8 (5.1)	19.8 (7.1)
Normal liver function (day)	14.7 (5.6)	14.6 (5.2)	15.8 (9.2)
Liver injury (day)	17.9 (5.5)	16.8 (4.7)	20.6 (6.4)
*p*	0.001	0.024	0.171
Duration of viral shedding (days)	12.3 (5.6)	11.4 (4.6)	15.8 (7.4)
Normal liver function (day)	10.5 (5.0)	10.5 (4.8)	10.8 (7.3)
Liver injury (day)	13.6 (5.7)	12.2 (4.4)	17.0 (7.0)
*p*	0.002	0.064	0.091

*Data are n (%) and mean (SD) unless specified otherwise*.

## Discussion

COVID-19 is a newly identified illness that has spread around the world and has become a global health crisis (2, 4, 7, 24). Epidemiological studies have demonstrated that liver injury can occur in patients with COVID-19 (7, 8, 10, 12) and may be related to SARS-CoV-2 infection or therapeutic drugs (9, 17). Here, the onset, progression, recovery, risk factors, and correlation with clinical outcomes of liver injury in patients with varying severities of COVID-19 were investigated. The results show that the liver injury in critically ill patients with COVID-19 occurred more frequently and earlier, developed more seriously, and recovered more slowly than that in non-critically patients. Drug factors, including the combination treatment of lopinavir/ritonavir and arbidol and the number of concomitant medications were independent risk factors for liver injury in non-critically ill patients with COVID-19, which may be due to drug interactions at the metabolic level. Liver injury was found to be related to prolonged hospital stay and delayed virus eradication in all enrolled patients.

Consistent with previous studies (7, 8, 25), this study has found that the high incidence of liver injury in patients with COVID-19 is related to disease severity. Additionally, the progression and the recovery process of liver injury during the entire hospital stay were investigated. Our results indicated that the liver injury in critically ill patients with COVID-19 happened earlier and recovered more slowly than that in non-critically ill patients. A higher rate of liver injury was presented in critically ill patients over the study period. Our further data showed that the severity of liver injury was also related to COVID-19 disease severity. The incidences of mild liver injury were similar in different groups, whereas moderate and severe liver injuries occurred more frequently in critically ill patients than in non-critically ill patients. SARS-CoV-2 may directly dysregulate liver function by binding to ACE2-positive cholangiocytes (26). Our results demonstrated that rather than TBL, ALT and AST were the most elevated indicators in critically ill patients with COVID-19. Hepatocyte injury can be caused by immune interactions that involve virally induced cytotoxic T and Kupffer cells (16). We speculated that besides the direct damage by SARS-CoV-2, virus-induced cytokine storm may also play an important role in critically ill patients with liver injury.

Particularly, unlike other studies, this study has found that drug factors rather than disease severity may play a more important role in the liver injury of non-critically ill patients with COVID-19. The combination treatment of lopinavir/ritonavir and arbidol was an independent risk factor. Lopinavir/ritonavir and arbidol are recommended as potential drugs for SARS-CoV-2 infection by the Chinese National Health Committee. Existing evidence shows that elevated serum aminotransferase and jaundice occur in patients receiving lopinavir/ritonavir containing antiretroviral regimens (18) and that arbidol may induce an increase in transaminase (27). Lopinavir and arbidol are mainly metabolized by cytochrome P3A (CYP3A) (28). Ritonavir is a potent CYP3A inhibitor. Our results demonstrated that lopinavir/ritonavir can significantly inhibit the metabolism of arbidol, thereby leading to increased arbidol serum concentration. In addition, TBL was the major elevated indicator in non-critically ill patients, indicating that arbidol may have novel adverse reactions in jaundice. Therapeutic drug monitoring may be useful in optimizing the regimens in COVID-19 patients receiving the combination treatment of lopinavir/ritonavir and arbidol. The number of concomitant medications was another independent predictor of liver injury. Concomitant drugs can affect the metabolism of other drugs through induction, inhibition, or substrate competition (20). We considered that an increased number of concomitant drugs may lead to complex drug interactions and can increase the risk of liver injury.

Our study also found that the liver injury in patients with COVID-19 was related to prolonged viral shedding and hospital stay durations. We inferred that liver injury would lead to immune dysfunction, thereby causing a delay in virus clearance. The prolonged hospital stay can be explained by the need for increased time for liver function recovery or the failure of virus eradication. In the critically ill group, the length of hospital stay and the duration of viral shedding in patients with liver injury were only numerically but not statistically higher than those in patients without liver injury. We speculated that the durations of hospital stay and viral shedding should be influenced by complex factors in critically ill patients with COVID-19, and liver injury may not be the only factor affecting the clinical outcomes.

Our study has some limitations. First, this study is a retrospective, non-randomized clinical observational trial. Our cohort is a convenience sample of patients with COVID-19 admitted to three hospitals in Zhejiang, China. However, this study reflects real-world clinical practices and provides relevant data about liver injury in patients with COVID-19. Second, the treatments among the three centers were highly consistent because all patients were from Zhejiang province. Antiviral agents were administered to all patients with COVID-19 but limited to a few kinds (e.g., lopinavir/ritonavir and arbidol). Therefore, our results cannot be extrapolated to antiviral agents that are not involved in this study, and large controlled studies are necessary to explore the potential risks of liver injury by other antivirals. Finally, univariable and multivariable logistic regression models were planned to be used to explore the risk factors for liver injury in the critically ill and non-critically ill groups. However, considering the limited sample size in the critically ill group, the statistical power was insufficient, and the multivariable logistic regression model was not conducted in this group. The trends in risk factors can be reflected through data comparison to a certain extent. Focusing on such a population with an expanded sample size would be challenging but would be interesting future research.

## Conclusion

Liver injury has occurred widely in patients with COVID-19. Critically ill patients suffered higher incidence, earlier occurrence, greater injury severity, and slower recovery from liver injury. Drug factors were independent risk factors for liver injury of non-critically ill patients, and drug interaction based on the CYP450 enzymes and concomitant drugs should be closely monitored. Liver injury was related to prolonged hospital stay and viral shedding duration in patients with COVID-19. Therefore, special attention to liver injury during SARS-CoV-2 infection is recommended. Healthcare workers should closely monitor the medications used during hospitalization and adjust and optimize the drug treatment in a timely manner.

## Data Availability Statement

All datasets presented in this study are included in the article/Supplementary Material.

## Ethics Statement

The studies involving human participants were reviewed and approved by the Ethics Committee of the First Affiliated Hospital, College of Medicine, Zhejiang University (Reference Number: 2020IIT[71]). Written informed consent for participation was not required for this study in accordance with the national legislation and the institutional requirements. This study followed the statement of Strengthening the Reporting of Observational Studies in Epidemiology.

## Author Contributions

TL, YQ, and XL participated in the conception and design of this study. SJ and XL were the project managers and coordinated patient recruitment. DH, LL, RW, RR, YR, JM, NC, XW, ZY, and MZ coordinated all analyses in the project. SJ, LL, RW, and DH were involved in the acquisition, analysis, or interpretation of data. MZ and YH conducted the *in vitro* experiments on drug interaction. Data analysis was done by DH, JM, ZY, and NC. Drafting of the manuscript was done by LL, RW, and XW. All authors contributed to the critical review and final approval of the manuscript.

## Conflict of Interest

The authors declare that the research was conducted in the absence of any commercial or financial relationships that could be construed as a potential conflict of interest.

## Abbreviations

COVID-19: Coronavirus disease 2019
SARS-CoV-2: Severe acute respiratory syndrome coronaviruses 2
DILI: Drug-induced liver injury
RUCAM: A Roussel Uclaf Causality Assessment Method
ALT: Alanine aminotransferase
AST: Aspartate aminotransferase
ALP: Alkaline phosphatase
TBL: Total bilirubin.

## References

[1] Li Q Guan X Wu P Wang X Zhou L Tong Y. Early transmission dynamics in Wuhan, China, of Novel Coronavirus-infected pneumonia. N Engl J Med. (2020) 382:1199–207. 10.1056/NEJMoa2001316 31995857 PMC7121484

[2] WHO Map production: WHO Health Emergencies Programme. (2020). Available online at: https://www.who.int/emergencies/diseases/novel-coronavirus-2019 (accessed May 30, 2020).

[3] Young BE Ong SW Kalimuddin S Low JG Tan SY Loh J. Epidemiologic features and clinical course of patients infected with SARS-CoV-2 in Singapore. JAMA. (2020) 323:1488–94. 10.1001/jama.2020.3204 32125362 PMC7054855

[4] Xu XW Wu XX Jiang XG Xu KJ Ying LJ Ma CL Clinical findings in a group of patients infected with the 2019 novel coronavirus (SARS-Cov-2) outside of Wuhan, China: retrospective case series. BMJ. (2020) 368:m606 10.1136/bmj.m606 32075786 PMC7224340

[5] Coronaviridae Study Group of the International Committee on Taxonomy of Viruses The species Severe acute respiratory syndrome-related coronavirus: classifying 2019-nCoV and naming it SARS-CoV-2. Nat Microbiol. (2020) 5:536–44. 10.1038/s41564-020-0695-z 32123347 PMC7095448

[6] WHO WHO Director-General's opening remarks at the media briefing on COVID-19 (2020). Available online at: https://www.who.int/dg/speeches/detail/who-director-general-s-opening-remarks-at-the-media-briefing-on-covid-19-−11-march-2020 (accessed May 30, 2020).

[7] Guan WJ Ni ZY Hu Y Liang WH Ou CQ He JX Clinical Characteristics of Coronavirus Disease 2019 in China. N Engl J Med. (2020) 382:1708–20. 10.1101/2020.02.06.20020974 32109013 PMC7092819

[8] Huang C Wang Y Li X Ren L Zhao J Hu Y. Clinical features of patients infected with 2019 novel coronavirus in Wuhan, China. Lancet. (2020) 395:497–506. 10.1016/S0140-6736(20)30183-5 31986264 PMC7159299

[9] Zhang C Shi L Wang FS. Liver injury in COVID-19 management and challenges. Lancet Gastroenterol Hepatol. (2020) 5:428–30. 10.1016/S2468-1253(20)30057-1 32145190 PMC7129165

[10] Chen N Zhou M Dong X Qu J Gong F Han Y. Epidemiological and clinical characteristics of 99 cases of 2019 novel coronavirus pneumonia in Wuhan, China: a descriptive study. Lancet. (2020) 395:507–13. 10.1016/S0140-6736(20)30211-7 32007143 PMC7135076

[11] Shi H Han X Jiang N Cao Y Alwalid O Gu J. Radiological findings from 81 patients with COVID-19 pneumonia in Wuhan, China: a descriptive study. Lancet Infect Dis. (2020) 20:425–34. 10.1016/S1473-3099(20)30086-4 32105637 PMC7159053

[12] Yang X Yu Y Xu J Shu H Xia J.a Liu H. Clinical course and outcomes of critically ill patients with SARS-CoV-2 pneumonia in Wuhan, China: a single-centered, retrospective, observational study. Lancet Respir Med. (2020) 8:475–81. 10.1016/S2213-2600(20)30079-5 32105632 PMC7102538

[13] Wang D Hu B Hu C Zhu F Liu X Zhang J. Clinical characteristics of 138 hospitalized patients with 2019 Novel Coronavirus-Infected Pneumonia in Wuhan, China. JAMA. (2020) 323:1061–9. 10.1001/jama.2020.1585 32031570 PMC7042881

[14] Fan Z Chen L Li J Tian C Zhang Y Huang S. Clinical features of COVID-19 related liver damage. Clin. Gastroenterol. Hepatol. (2020) 18:1561–66. 10.1101/2020.02.26.20026971 32283325 PMC7194865

[15] Xu L Liu J Lu M Yang D Zheng X. Liver injury during highly pathogenic human coronavirus infections. Liver Int. (2020) 40:998–1004. 10.1111/liv.14435 32170806 PMC7228361

[16] Bangash MN Patel J Parekh D. COVID-19 and the liver: little cause for concern. Lancet Gastroenterol Hepatol. (2020) 5:529–30. 10.1016/S2468-1253(20)30084-4 32203680 PMC7270582

[17] Xu Z Shi L Wang Y Zhang J Huang L Zhang C. Pathological findings of COVID-19 associated with acute respiratory distress syndrome. Lancet Respir Med. (2020) 8:420–2. 10.1016/S2213-2600(20)30076-X 32085846 PMC7164771

[18] LiverTox: Clinical Research Information on Drug- Induced Liver Injury [Internet] Bethesda (MD): National Institute of Diabetes Digestive Kidney Diseases (2020). Available online at: https://www.ncbi.nlm.nih.gov/books/NBK547852/#IX-L (accessed March 1, 2020). 31643176

[19] Al-Aqil FA Monte MJ Peleteiro-Vigil A Briz O Rosales R González R. Interaction of glucocorticoids with FXR/FGF19/FGF21-mediated ileum-liver crosstalk. Biochim Biophys Acta Mol Basis Dis. (2018) 1864:2927–37. 10.1016/j.bbadis.2018.06.003 29883717

[20] Andrade RJ Aithal GP Björnsson ES Kaplowitz N Kullak-Ublick GA Larrey D. EASL Clinical Practice guidelines: drug-induced liver injury. J Hepatol. (2019) 70:1222–61. 10.1016/j.jhep.2019.02.014 30926241

[21] Leise MD Poterucha JJ Talwalkar JA. Drug-induced liver injury. Mayo Clin Proc. (2014) 89:95–106. 10.1016/j.mayocp.2013.09.016 24388027

[22] National Health Commission and State Administration of Traditional Chinese Medicine Diagnosis and Treatment Protocol for Novel Coronavirus Pneumonia (Trial Version 6). (2020). Available online at: http://www.nhc.gov.cn/yzygj/s7653p/202002/8334a8326dd94d329df351d7da8aefc2.shtml (accessed May 1, 2020). 10.1097/IM9.0000000000000022 PMC7227203 40479241

[23] NCI CTCAE. Available online at: http://evs.nci.nih.gov/ftp1/CTCAE/About.html (accessed March 1, 2020).

[24] WHO Coronavirus Disease (COVID-19):Outbreak. (2020). Available online at: https://www.who.int/emergencies/diseases/novel-coronavirus-2019 (accessed May 30, 2020).

[25] Cai Q Huang D Ou P Yu H Zhu Z Xia Z. COVID-19 in a designated infectious diseases hospital outside Hubei Province, China. Allergy. (2020). 10.1101/2020.02.17.20024018. [Epub ahead of print]. 32239761

[26] Chai X Hu L Zhang Y Han W Lu Z Ke A pecific ACE2 expression in cholangiocytes may cause liver damage after 2019-nCoV infection. bioRxiv. (2020). 10.1101/2020.02.03.931766. [Epub ahead of print].

[27] Meng-zhao W Long-yun CB Nan LJ Hong-xia Y He G Jian-zhong Z. Efficacy and safety of arbidol in treatment of naturally acquired influenza. Zhongguo Yi Xue Ke Xue Yuan Xue Bao. (2004) 26:289–93. 15266832

[28] Deng P Zhong D Yu K Zhang Y Wang T Chen X. Pharmacokinetics, metabolism, and excretion of the antiviral drug arbidol in humans. Antimicrob Agents Chemother. (2013) 57:1743–55. 10.1128/AAC.02282-12 23357765 PMC3623363

