# Supplementary material for: Liver Injury in Critically Ill and Non-critically Ill COVID-19 Patients: A Multicenter, Retrospective, Observational Study
Source: Front Med (Lausanne). 2020 Jun 23;7:347. doi: 10.3389/fmed.2020.00347 (PMC7324794; doi:10.3389/fmed.2020.00347)
Supplement: Supplementary file 1 [file Data_Sheet_1.docx]

**Liver injury in critically ill and non-critically ill COVID-19 patients: a multicenter, retrospective, observational study**

**Supplementary Materials**

**Supplementary Table S1 Characteristics of critically ill patients with or without liver injury**

| **Characteristics** | **Patients without liver injury (n=5)** | **Patients with liver injury (n=22)** | ***p*** |
| --- | --- | --- | --- |
| **Age, Years** |  |  |  |
| Mean | 68.5 (16.1) | 64.7 (16.9) | 0.563 |
| Range | 54–96 | 37–91 |  |
| **Sex** |  |  | 0.295 |
| Male | 2 (40.0%) | 16 (72.7%) |  |
| Female | 3 (60.0%) | 6 (27.3%) |  |
| **BMI** | 23.7 (3.9) | 24.2 (2.1) | 0.714 |
| **Chronic medical illness** |  |  |  |
| Cardiovascular and cerebrovascular diseases | 5 (100.0%) | 15 (68.2%) | 0.283 |
| Endocrine system disease | 0 (0.0%) | 8 (36.4%) | ·· |
| Digestive system disease | 0 (0.0%) | 2 (9.1%) | ·· |
| **Laboratory results** |  |  |  |
| Leucocytes (×10^9^/L) | 10.7 (6.6) | 9.0 (5.0) | 0.475 |
| Neutrophils (%) | 73.3 (36.4) | 78.7 (27.3) | 0.677 |
| Lymphocytes (×10^9^/L) | 1.4 (2.4) | 0.64 (0.45) | 0.346 |
| Haemoglobin (g/L) | 130.9 (17.2) | 133.0 (17.4) | 0.763 |
| Platelets (×10^9^/L) | 169.5 (78.0) | 172.6 (45.9) | 0.909 |
| C-reactive protein (mg/L) | 47.2 (33.4) | 51.0 (48.3) | 0.808 |
| Total bilirubin (μmol/L) | 11.9 (6.4) | 11.7 (5.2) | 0.909 |
| Direct bilirubin (μmol/L) | 5.8 (2.8) | 6.6 (3.9) | 0.555 |
| Alanine aminotransferase (ALT, μmol/L) | 18.1 (7.6) | 21.7 (9.2) | 0.268 |
| Aspartate aminotransferase (AST, μmol/L) | 23.7 (7.4) | 28.0 (9.4) | 0.194 |
| Alkaline phosphatase (ALP, U/L) | 62.3 (20.8) | 62.9 (21.7) | 0.943 |
| Albumin (g/L) | 32.5 (9.7) | 36.1 (5.3) | 0.274 |
| Serum creatinine (μmol/L) | 80.6 (21.8) | 102.1 (80.9) | 0.317 |
| **Number of concomitant medications** | 14 (IQR, 11–23) | 18 (IQR, 15–28) | 0.232 |
| **Concomitant medications** |  |  |  |
| Glucocorticoids | 5 (100.0%) | 21 (95.5%) | 1 |
| Quinolones | 1 (20.0%) | 6 (27.3%) | 1 |
| NSAIDs | 0 (0.0%) | 2 (9.1%) | .. |
| **Antiviral agents** |  |  |  |
| Lopinavir/ritonavir + arbidol^a^ | 2 (40.0%) | 11 (50.0%) | 1 |
| Darunavir/cobicistat-based therapy^b^ | 2 (40.0%)) | 10 (45.5%) | 1 |
| Others | 1 (20.0%) | 1 (4.5%) | 0.342 |

Data are n (%) and mean (SD) unless specified otherwise.

**Supplementary Table S2. Characteristics of non-critically ill patients with or without liver injury**

|  | **Patients without liver injury（n=50)** | **Patients with liver injury（n=54)** | ***p*** |
| --- | --- | --- | --- |
| **Age, Years** |  |  |  |
| Mean | 48.7 (12.3) | 45.7 (14.2) | 0.250 |
| Range | 24–85 | 19–88 |  |
| **Sex** |  |  | 0.239 |
| Male | 22 (44.0%) | 30 (55.6%) |  |
| Female | 28 (56.0%) | 24 (44.4%) |  |
| **BMI** | 23.2 (3.0) | 23.2 (3.5) | 0.992 |
| **Chronic medical illness** |  |  |  |
| Cardiovascular and cerebrovascular diseases | 11 (22.0%) | 6 (11.1%) | 0.134 |
| Endocrine system disease | 8 (16.0%) | 6 (11.1%) | 0.465 |
| Digestive system disease | 2 (4.0%) | 1 (1.9%) | 0.607 |
| Neurological disorders | 3 (6.0%) | 1 (1.9%) | 0.349 |
| Immune system | 1 (2.0%) | 1 (1.9%) | 1 |
| **Laboratory results** |  |  |  |
| Leucocytes (×10^9^/L) | 5.8 (2.8) | 6.1 (3.2) | 0 .507 |
| Neutrophils (%) | 68.3 (14.4) | 67.7 (16.3) | 0.853 |
| Lymphocytes (×10^9^/L) | 1.8 (3.5) | 2.7 (6.5) | 0.401 |
| Haemoglobin (g/L) | 133.5 (17.4) | 140.0 (18.3) | 0.066 |
| Platelets (×10^9^/L) | 221.8 (88.0) | 196.7 (64.5) | 0.098 |
| C-reactive protein (mg/L) | 23.2 (35.2) | 17.4 (30.3) | 0.370 |
| Total bilirubin (μmol/L) | 10.3 (5.5) | 13.6 (9.7) | 0.033 |
| Direct bilirubin (μmol/L) | 4.6 (2.2) | 5.9 (4.2) | 0.047 |
| Alanine aminotransferase (ALT, μmol/L) | 22.2 (18.5) | 25.8 (14.6) | 0.273 |
| Aspartate aminotransferase (AST, μmol/L) | 23.2 (10.9) | 26.4 (10.3) | 0.129 |
| Alkaline phosphatase (ALP, U/L) | 70.5 (17.5) | 66.1 (14.8) | 0.170 |
| Albumin (g/L) | 40.1 (5.5) | 40.9 (4.4) | 0.423 |
| Serum creatinine (μmol/L) | 69.9 (20.5) | 69.2 (19.1) | 0.865 |
| **Disease severity** |  |  |  |
| Severe | 13 (26.0%) | 16 (29.6%) | 0.680 |
| **Number of concomitant medications** | 7 (IQR, 4–9) | 9 (IQR, 6–13) | 0.018 |
| **Concomitant medications** |  |  |  |
| Glucocorticoids | 18 (36.0%) | 22 (40.7%) | 0.620 |
| Quinolones | 10 (20.0%) | 13 (24.1%) | 0.617 |
| NSAIDs | 3 (6.0%) | 4 (7.4%) | 1 |
| Statins | 3 (6.0%) | 1 (1.9%) | 0.349 |
| Immunosuppressive agents | 1 (2.0%) | 2 (3.7%) | 1 |
| **Antiviral agents** |  |  |  |
| Lopinavir/ritonavir + arbidol | 40 (80.0%) | 50 (92.6%) | 0.060 |
| Darunavir/cobicistat-based therapy | 6 (12.0%) | 2 (3.7%) | 0.150 |
| Others | 4 (8.0%) | 2 (3.7%) | 0.424 |

Data are n (%) and mean (SD) unless specified otherwise.
